# Benchmarking healing times for diabetic foot ulcerations and investigating the influence of peripheral arterial disease and infection

**DOI:** 10.1186/1757-1146-4-S1-O31

**Published:** 2011-05-20

**Authors:** Tamara E Milne, Deborah E Schoen, Virginia M Bower, Joel M Gurr

**Affiliations:** 1 Department of Podiatry, Royal Perth Hospital, Perth, WA, 6001, Australia; 2 University of Western Australia, Crawley, WA, 6008, Australia

## Background

To calculate benchmark healing times for diabetic foot ulcerations (DFU) seen in a tertiary hospital Podiatry Department, and investigate the influence of peripheral arterial disease (PAD) and infection (soft tissue infection [STI] and osteomyelitis [OM]) on healing times.

## Methods

Data was collected prospectively between October 2004 and September 2008 for all patients with diabetes who presented with a DFU. All DFU that healed within the time frame of the study were included. Data was collected for the following variables: presentation date, healing date, gender, location, presence of peripheral neuropathy (PN), presence of PAD, presence of infection (STI or OM), and whether the DFU was reviewed on the Multidisciplinary Foot Ulcer Clinic (MDFUC).

## Results

A total of 623 healed DFU were recorded (73% male) and analysed. 67% of DFU were exclusively neuropathic. PAD was diagnosed in 30.2% of DFU. Infection presented in 44% of DFU (31% STI and 13% OM). The median healing time of all DFU was 52 days. The median healing time of DFU complicated by PAD was 57 days compared to 49 days for DFU without PAD (p= 0.039). DFU complicated by STI and OM had median healing times of 83 days and 126 days respectively compared to 34 days for DFU without infection (p=0.0001).

## Conclusions

This research shifts the focus away from healing rates and outcomes to provide preliminary data for healing times of DFU. The data provides a benchmark for comparison to other tertiary hospitals and health services managing DFU, with an aim to ultimately aid in facilitating optimal clinical outcomes. Importantly, the data from our study emphasises and quantifies the detrimental influence that infection and PAD has on the healing time required for DFU, and ultimately hospital resources.

